# F107. CSF ABNORMALITIES IN SCHIZOPHRENIA AND DEPRESSION: PRELIMINARY RESULTS FROM A LARGE SCALE COHORT

**DOI:** 10.1093/schbul/sby017.638

**Published:** 2018-04-01

**Authors:** Tatiana Oviedo Salcedo, Anna Gagsteiger, Lot de Witte, Tania Kümpfel, René Kahn, Peter Falkai, Peter Eichhorn, Jurjen Luykx, Alkomiet Hasan

**Affiliations:** 1 Ludwig Maximilian University; 2 University Medical Center Utrecht, Brain Center Rudolf Magnus

## Abstract

**Background:**

CSF abnormalities and a neuroinflammatory pathophysiology have been discussed for affective and non-affective psychosis for more than 30-years. Recent studies pointed towards a specific phenotype of autoimmune-antibody mediated psychosis, but evidence is still sparse. Especially CSF data investigating autoimmune antibodies in large-scale CSF cohorts of affective and non-affective psychoses are lacking.

**Methods:**

We analyzed a retrospective naturalistic cohort of 592 patients with A) schizophrenia-spectrum disorders (N = 330) or B) depressive disorders (N = 262) who underwent a lumbar puncture as part of the clinical routine in the Department of Psychiatry and Psychotherapy at the Ludwig-Maximilians University Munich between July 2012 and May 2017. We used a predefined systematic algorithm for the database search in the clinical documentation system and data was extracted by TO and AG. The study was approved by the local ethics committee.

**Results:**

We identified 592 patients with standard CSF parameters. Schizophrenia spectrum patients did not differ from depressive patients with regard to the white blood cell count (cells/µl) (p = 0.774) or albumin quotient (p = 0.663). The general prevalence of oligoclonal bands did not differ between groups (schizophrenia: 37.0%, depression: 37.8%; p = 0.838). However, schizophrenia patients showed higher frequencies for intrathecal oligoclonal bands (32% of all oligoclonal bands) compared to depressive patients (19.1% of all oligoclonal bands. (p = 0.034). 124 schizophrenia-spectrum patients (54 first-episode patients) received CSF analyses for neural antibodies. None of the patients showed positive CSF results in any of the tested autoimmune-encephalitis panel (NMDA(N=119), AMPA-1(N=114), AMPA-2(N=114), CASPR(N=111), LGI-1(N=110) and GABA-B(N=112)-Antibodies) in CSF. The results for the intracellular onconeuronal and synaptic antibodies were also negative (Amphyphysin(N=93), Yo(N=58), Hu(N=94), Ri(N=94), CV2(N=93), Ma1(N=93) and Ma2(N=93)-Antibodies). Three of these patients with negative CSF titers did have low-titer neuronal antibodies in serum: CASPR-2-AB: 1:10, CASPR-2-AB: 1:50, Yo-AB: low band-intensity. 36 depression patients were also tested for autoimmune antibodies and again no positive reports could be identified in CSF.

**Discussion:**

This is the first analyses of autoimmune antibodies in first-episode and recurrent schizophrenia and depressive mood disorder showing no positive CSF titers. However, schizophrenia patients have a higher prevalence of intrathecal oligoclonal bands compared to affective patients pointing towards more immunological disturbances in this population. The here presented analyses are exploratory and need to undergo confirmatory analyses and quality control.

